# A Unique Role of GATA1s in Down Syndrome Acute Megakaryocytic Leukemia Biology and Therapy

**DOI:** 10.1371/journal.pone.0027486

**Published:** 2011-11-16

**Authors:** Ana C. Xavier, Holly Edwards, Alan A. Dombkowski, Tugce B. Balci, Jason N. Berman, Graham Dellaire, Chengzhi Xie, Steven A. Buck, Larry H. Matherly, Yubin Ge, Jeffrey W. Taub

**Affiliations:** 1 Division of Pediatric Hematology/Oncology, Children's Hospital of Michigan, Detroit, Michigan, United States of America; 2 Department of Oncology, Wayne State University School of Medicine, Detroit, Michigan, United States of America; 3 Developmental Therapeutics Program, Barbara Ann Karmanos Cancer Institute, Wayne State University School of Medicine, Detroit, Michigan, United States of America; 4 Division of Pharmacology and Toxicology, Children's Hospital of Michigan, Detroit, Michigan, United States of America; 5 Department of Pediatrics, Wayne State University School of Medicine, Detroit, Michigan, United States of America; 6 IWK Health Centre, Department of Pediatrics, Dalhousie University, Halifax, Canada; 7 Department of Biochemistry & Molecular Biology, Dalhousie University, Halifax, Canada; 8 Department of Pharmacology, Wayne State University School of Medicine, Detroit, Michigan, United States of America; Instituto Nacional de Câncer, Brazil

## Abstract

**Background:**

Acute megakaryocytic leukemia (AMkL) in Down syndrome (DS) children is uniformly associated with somatic *GATA1* mutations, which result in the synthesis of a shorter protein (GATA1s) with altered transactivation activity compared to the wild-type GATA1. It is not fully established whether leukemogenesis and therapeutic responses in DS AMkL patients are due to loss of the wild-type GATA1 or due to a unique function of GATA1s.

**Methodology:**

Stable clones of CMK cells with decreased GATA1s or Bcl-2 levels were generated by using *GATA1-* or *BCL-2*-specific lentivirus shRNAs. *In vitro* ara-C, daunorubicin, and VP-16 cytotoxicities of the shRNA stable clones were determined by using the Cell Titer-blue reagent. Apoptosis and cell cycle distribution were determined by flow cytometry analysis. Changes in gene transcript levels were determined by gene expression microarray and/or real-time RT-PCR. Changes in protein levels were measured by Western blotting. *In vivo* binding of GATA1s to *IL1A* promoter was determined by chromatin immunoprecipitation assays.

**Results:**

Lentivirus shRNA knockdown of the *GATA1* gene in the DS AMkL cell line, CMK (harbors a mutated *GATA1* gene and only expresses GATA1s), resulting in lower GATA1s protein levels, promoted cell differentiation towards the megakaryocytic lineage and repressed cell proliferation. Increased basal apoptosis and sensitivities to ara-C, daunorubicin, and VP-16 accompanied by down-regulated Bcl-2 were also detected in the CMK *GATA1* shRNA knockdown clones. Essentially the same results were obtained when Bcl-2 was knocked down with lentivirus shRNA in CMK cells. Besides Bcl-2, down-regulation of GATA1s also resulted in altered expression of genes (e.g., *IL1A, PF4, and TUBB1*) related to cell death, proliferation, and differentiation.

**Conclusion:**

Our results suggest that GATA1s may facilitate leukemogenesis and potentially impact therapeutic responses in DS AMkL by promoting proliferation and survival, and by repressing megakaryocytic lineage differentiation, potentially by regulating expression of Bcl-2 protein and other relevant genes.

## Introduction

Down Syndrome (DS) children with leukemia exhibit some of the most unique biological and therapeutic features of leukemia. DS children have an estimated 10-20-fold higher risk for developing acute lymphoblastic leukemia and acute myeloid leukemia (AML) compared to non-DS children.[1] The majority of AML cases in DS children are of the acute megakaryocytic leukemia (AMkL) phenotype.[2]–[5] It is estimated that DS children have a 500-fold increased risk of developing AMkL compared to non-DS children.[6] Transient myeloproliferative disorder (TMD), a precursor of AMkL, is diagnosed in up to 10% of DS newborns and can resolve spontaneously without chemotherapy in the majority of cases.[7] It is estimated that following clinical resolution, 20–30% of DS TMD patients will subsequently develop AMkL, requiring chemotherapy treatment.[8] Multiple clinical trials have shown that DS children with AML, and in particular AMkL, have extremely high event-free survival (EFS) rates (∼80–100%) when treated with cytosine arabinoside (ara-C)/anthracycline-based chemotherapy.[2]–[5], [9] This is in marked contrast to the ∼50% EFS rates typically seen for non-DS pediatric AML patients, and the <35% EFS rates for non-DS pediatric AMkL patients.[2]–[5], [9]

What are the molecular bases for the increased incidence of leukemia in DS children, and in particular, TMD and AMkL? Acquired somatic mutations of the transcription factor gene *GATA1* (localized to Xp11.23) have been consistently detected in nearly all DS TMD and AMkL cases, while mutations have not been detected in DS acute lymphoblastic leukemia and non-DS AML and AMkL except for rare cases.[10]–[12] *GATA1* gene encodes a zinc finger transcription factor that binds to the WGATAR motif and is essential for normal erythroid and megakaryocytic differentiation.[13] The net effect of the mutations is the introduction of stop codons either before or after methionine 84 that results in a 40-kDa truncated GATA1 protein (designated GATA1s), initiated from a downstream translation start site and distinguishable from GATA1 (50-kDa).[12] Both GATA1s and GATA1 show similar DNA binding abilities and interact with partner proteins, such as “Friend of GATA1” (FOG1), though GATA1s exhibits altered transactivation capacity due to the loss of the *N*-terminal activation domain.[12] This could potentially contribute to the uncontrolled proliferation of poorly differentiated megakaryocytic precursors. In fact, GATA1s has been shown to lead to hyperproliferation of a unique, previously unrecognized yolk sac and fetal liver progenitor in transgenic mice, which may account for the transient nature of TMD in DS.[14]

The uniform detection of somatic *GATA1* gene mutations in DS AMkL cases suggests that loss of the wild-type GATA1 and/or synthesis of GATA1s in DS AMkL may somehow contribute to the high EFS rates of DS AMkL patients. Indeed, a relationship between GATA1 and AML outcome was suggested by a Japanese clinical study in which non-DS AML patients with lower *GATA1* transcript expression experienced the highest complete remission rates.[15] In our previous study, when the wild-type GATA1 was ectopically over-expressed in a DS AMkL cell line, CMK (harbors a mutated *GATA1* gene and only expresses GATA1s), it resulted in significantly increased resistance to ara-C, suggesting that loss of GATA1 could be responsible for the enhanced therapeutic responses of DS AMkL patients.[16] However, it is not clear whether the enhanced therapeutic responses of DS AMkL patients are due primarily to loss of the wild-type GATA1 and/or due to unique biological functions of GATA1s in DS AMkL cases.

To date, no studies have been reported to determine the role of GATA1s in a human DS AMkL cell line model. In this study, we explored the functional role of GATA1s in DS AMkL biology and therapy using lentivirus shRNA to knockdown *GATA1* in the DS AMkL cell line, CMK, which expresses only GATA1s and no wild-type GATA1.[17] Our results suggest that GATA1s has unique functions in facilitating DS leukemogenesis and in modulating therapeutic responses by repressing differentiation towards the megakaryocytic lineage, and by promoting proliferation and survival, potentially through regulating expression of Bcl-2 and other relevant genes.

## Materials and Methods

### Cell Culture

The DS AMkL cell line, CMK, was obtained from the German Collection of Microorganisms and Cell Cultures (DSMZ; Braunschweig, Germany). The parental CMK cells and the shRNA stable clones were cultured in RPMI 1640 with 10% fetal bovine serum (Hyclone, Logan, UT) and 2 mM L-glutamine plus 100 U/ml penicillin and 100 µg/ml streptomycin, in a 37°C humidified atmosphere containing 5% CO_2_/95% air.

### Lentiviral ShRNA Knockdown of GATA1 and Bcl-2 in CMK Cells

Knockdown of *GATA1* and *Bcl-2* genes in the CMK cells was performed using shRNA lentivirus (Sigma-Aldrich, St. Louis, MO), as previously reported.[18], [19] The sequences for the negative control, *GATA1*, and *Bcl-2* shRNAs are shown in Figure S1. Two clones for each gene (designated CMK-5a and CMK-5b for *GATA1*, and CMK-b7 and CMK-b8 for *Bcl-2*) with decreased expression of GATA1s and Bcl-2, respectively, were selected for further studies. A pool of cells from the negative control infection (lentivirus expressing a shRNA with limited homology to any known human genes) was used as the negative control (designated CMK-neg) for the *GATA1* and *Bcl-2* shRNA stable clones.

### Cell Differentiation and Proliferation Assays

The effects of GATA1s on megakaryocytic differentiation were assessed by flow cytometry analysis of cell surface markers of the CMK-neg, -5a, and -5b stable clones, as previously described.[20] To determine the effects of GATA1s on cell proliferation, the CMK-neg, -5a, and -5b stable clones were seeded at 2.5×10^4^ cells/mL in T-25 flasks and counted every 24 hours to determine doubling times for each stable clone. To verify possible changes in cell cycle progression, CMK-5a, -5b, and –neg cells were harvested and fixed with ice-cold 70% (v/v) ethanol for 24h. After centrifugation at 200×g for 5 min, the cell pellets were washed with phosphate-buffered saline (PBS, pH 7.4) and resuspended in PBS containing propidium iodine (PI) (50 µg/mL), triton X-100 (0.1%, v/v), and DNase-free RNase (1 µg/mL). The DNA contents were determined by flow cytometry using a FACScan flow cytometer (BD Biosciences, San Jose, CA). Cell cycle analysis was done with the Multicycle software (Phoenix Flow Systems, Inc.).

### BrdU Labeling and Flow Cytometric Analysis

The CMK-neg, -5a, and -5b cells were cultured in flasks (2−5×10^5^/ml) and incubated for 1 hour with 10 µM BrdU (5-Bromo-2′-deoxyuridine, Sigma-Aldrich, St. Louis, MO). Cells were harvested, washed, fixed and treated with 2 N HCl. After washing with PBS, cells were blocked with 0.2% Tween-20 in PBS and incubated with fluorescein isothiocyanate (FITC) conjugated anti-BrdU primary antibody (BD Pharmingen FITC Mouse Anti-BrdU Set, San Diego, CA) in the same buffer for 2 hours at room temperature. Cells were washed and resuspended in PBS with 2 µg/mL 7-AAD (Sigma-Aldrich, St. Louis, MO) and subsequently submitted to flow cytometry. Flow cytometric analysis was done on at least 10,000 cells from each sample, using a FACS Calibur flow cytometer (BD BioSciences). Three independent experiments were completed.

### In Vitro Ara-C Cytotoxicity Assays

For determinations of *in vitro* ara-C cytotoxicities, the CMK stable clones were cultured in complete medium with dialyzed fetal bovine serum (FBS) in 96-well plates at a density of 8×10^4^ cells/mL. Cells were cultured for 96 h with a range of concentrations of ara-C at 37°C, and viable cell numbers were determined using the Cell Titer-blue reagent (Promega, Madison, WI) and a fluorescence microplate reader. The IC_50_ values were calculated as the concentrations of ara-C necessary to inhibit 50% proliferation compared to untreated control cells. The data are presented as mean values ± standard errors from a minimum of 3 independent experiments.

### Assessment of Baseline Apoptosis

The CMK shRNA stable clones were harvested, vigorously pipetted, and triplicate samples were used for determining baseline apoptosis with the Apoptosis Annexin-V FITC/PI Kit (Beckman Coulter; Brea, CA), as previously described.[18], [19] Apoptotic events were recorded as a combination of Annexin-V+/PI- (early apoptotic) and Annexin-V+/PI+ (late apoptotic/dead) events.

### Western Blot Analysis

Soluble protein extracts were prepared by sonication in hypotonic buffer (10 mM Tris-Cl, pH 7.0), containing 1% SDS and proteolytic inhibitors, and subjected to SDS-PAGE. Separated proteins were electrophoretically transferred to PVDF membranes (Thermo Fisher Inc., Rockford, IL) and immunoblotted with anti-GATA1, -Bax, -Bad, -Bid, -Bcl-xL, –Bcl-2 (Cell Signaling Technology, Danvers, MA), or -β-actin (Sigma-Aldrich, St. Louis, MO) antibodies, as described previously.[16] Immunoreactive proteins were visualized using the Odyssey Infrared Imaging System (Li-Cor, Lincoln, NE), as described by the manufacturer.

### Gene Expression Microarray Analysis

Gene expression microarray was performed with the Agilent Whole Human Genome 4x44K microarray, catalog #G4112F. Microarray sample preparation, hybridization, and data analysis were described previously.[20] On each microarray, a labeled CMK-5a or -5b sample was co-hybridized with an oppositely labeled CMK-neg sample. Two arrays were completed for the CMK-5a/CMK-neg pair and the CMK-5b/CMK-neg pair, respectively, for a total of four arrays. The two microarrays used for each clone were hybridized in a dye swap arrangement with opposite dye orientation to minimize the dye bias effect. Statistical analyses were performed using Rosetta Resolver®.[21] We have deposited the raw data at GEO under accession number GSE32388, we confirm all details are MIAME compliant.

To identify overlapping probes/genes between the CMK gene set and our previously reported gene set derived from a comparison between DS and non-DS AMkL primary patient samples,[22] the probe IDs of each gene set were cross referenced to Entrez Gene IDs, and a common set of Entrex IDs between the 2 groups was identified.

### Quantification of Gene Expression by Real-time RT-PCR

Transcripts for *GATA1*, *Bcl-2*, *deoxycytidine kinase (dCK)*, *cytidine deaminase (CDA)*, *human equilibrative nucleoside transporter 1 (hENT1)*, *interleukin 1 alpha (IL1A)*, *platelet factor 4 (PF4)*, and *tubulin, beta 1(TUBB1)* were quantitated using a LightCycler real-time PCR machine (Roche, Indianaopolis, IN), as previously described,[16] or using Taqman probes (Applied Biosystems Inc, Foster City, CA, for *PF4* and *TUBB1*). Primers and PCR conditions are described in Table S1. Real-time PCR results were expressed as mean values ± standard errors from 3 independent experiments using the same cDNA preparation and normalized to *GAPDH* (*glyceraldehyde 3-phosphate dehydrogenase*) transcripts.

### Chromatin Immunoprecipitation Assays

The chromatin immunoprecipitation (ChIP) assay was performed as described previously.[23], [24] Purified chromatin fragments from CMK cells were incubated with anti-GATA1 antibody or normal IgG. Standard PCR (qualitative) for the GATA1 binding region in the *IL1A* promoter was performed with forward (5′-GGGGAATTATTTACAACAGAGGAGTG-3′) and reverse (5′-TAATGCCTCAGTTCACCAAAGAAA-3′) primers spanning the three putative GATA1 binding sites. An upstream unrelated (ie, to GATA1 binding) region in the *IL1A* gene was amplified by PCR with the use of upper (5′-CTACATCAATCACCCATCTCCA-3′) and lower (5′-CTTCACGTTCAGTCAGCAAAT-3′) primers to further validate the specificity of the ChIP assays. The same *IL1A* promoter regions were also amplified by real-time PCR (quantitative), using the same primers, on a Roche LightCycler-480 real-time PCR machine.

### Effects of Recombinant IL1α on Cell Proliferation of CMK-5a Cells

CMK-5a cells were seeded at a density of 2.5×10^4^ cells/mL in T25 flasks and either recombinant IL1α (ProSpec, Rehovot, Israel) reconstituted in PBS, or an equal volume of PBS was added. The cells were cultured for up to 48 h at 37°C and counted in triplicate every 24 h.

### Statistical Analysis

Student's t test (two tail, unequal variance) was used for statistical analysis on experiments unless otherwise specified.

## Results

### GATA1s Promotes Cell Proliferation and Represses Megakaryocytic Differentiation of CMK Cells

**Figure 1:**
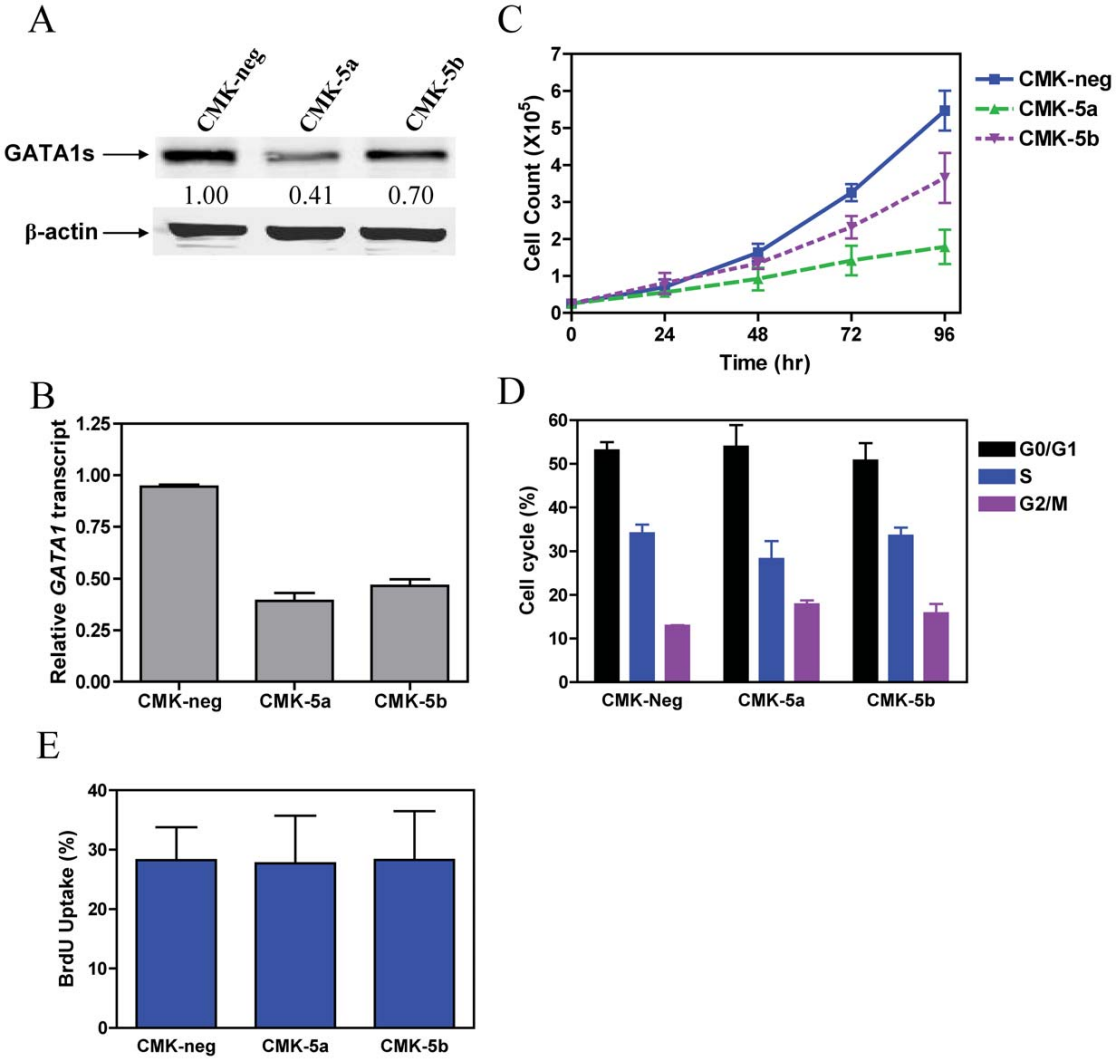
Down-regulation of GATA1s in CMK cells results in impaired cell proliferation. Expression of GATA1s in two selected subclones, CMK-5a and -5b, in comparison to the negative control (CMK-neg) was verified by Western blotting (**panel A**) and real-time RT-PCR (**panel B**). The real-time RT-PCR results were expressed as mean values ± standard errors from 3 independent experiments using the same cDNA preparation and normalized to GAPDH. To establish the doubling times for each shRNA subclone, the CMK-5a, -5b, and –neg sublines were seeded at 2.5×10^4^ cells/ml and counted every 24 h with trypan blue staining (**panel C**). Cell cycle progression in the CMK-5a, -5b, and –neg sublines was assessed by PI staining and flow cytometry analysis, as described in the Materials and Methods (**panel D**). DNA content was also assessed in the CMK-5a, -5b, and –neg sublines by incorporating BrdU into DNA and flow cytometry analysis as described in the Materials and Methods (**panel E**).

To investigate the role of GATA1s in DS AMkL, we used lentiviral shRNA to knockdown the *GATA1* gene in a DS AMkL cell line, CMK, established from a 1-year-old DS boy with AMkL and harboring a mutated *GATA1* gene.[17] shRNA lentiviral infection resulted in 59% and 30% decreased levels of GATA1s in two clones, designated CMK-5a and -5b, respectively, compared to a pool of cells from the negative control infection (designated CMK-neg, Fig. 1A). This was accompanied by parallel changes in transcript levels for *GATA1* in the CMK-5a and -5b cells, compared to the CMK-neg cells (Fig. 1B). Down-regulation of GATA1s was accompanied by GATA1s dose-dependent decreased proliferation of the CMK-5a and -5b stable clones (doubling times 36.4 and 28.2 h, respectively), compared to the CMK-neg cells (25.8 h, Fig. 1C). Interestingly, this was unlikely related to cell cycle perturbations since only minimal changes of the cell cycle phases (e.g., slight decreases in number of cells in S phase) in the CMK-5a and -5b clones were detected by PI staining and flow cytometry analyses, compared to the CMK-neg cells (Fig. 1D). The lack of obvious changes in S phase in the CMK *GATA1* shRNA stable clones was further confirmed by BrdU incorporation assays (Fig. 1E). These results demonstrate that GATA1s promotes cell proliferation in DS AMkL.

**Figure 2:**
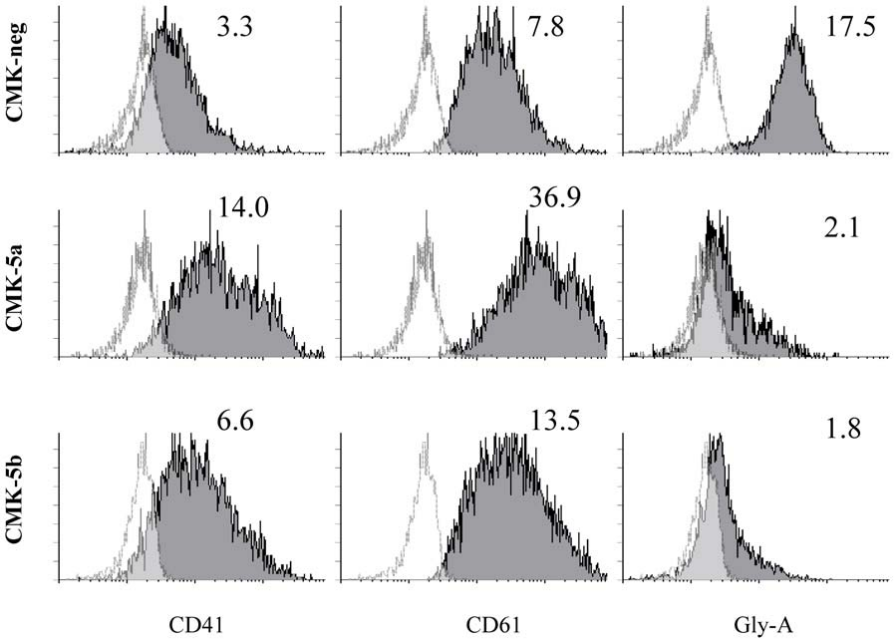
Down-regulation of GATA1s in CMK cells results in increased differentiaion toward megakaryocytic lineage. Megakaryocytic cell surface marker expression in the CMK-5a, -5b, and -neg stable clones was determined by flow cytometry. The unshaded plot represents isotype control. Numbers (isotype ratio) represent the ratio between the quantitative expression value of the cell surface marker and isotype control.

To determine the role of GATA1s in megakaryocytic differentiation, flow cytometry analysis of megakaryocytic-associated antigens (e.g., CD61 and CD41) on the cell surface was performed in the CMK-5a, -5b, and –neg cells. Interestingly, down-regulation of GATA1s in CMK cells resulted in a significantly increased total cell surface expression of CD61 (isotype ratio of 36.9 and 13.5, respectively) and CD41 (isotype ratio of 14 and 6.6, respectively) as a measure of megakaryocyte maturation in the CMK-5a and CMK-5b clones relative to the CMK-neg cells (isotype ratio of 7.8 and 3.3, respectively; p<0.01) (Fig. 2). Total cell surface expression of glycophorin-A was significantly decreased in the CMK-5a and CMK-5b clones (isotype ratio of 2.1 and 1.8, respectively), compared to the CMK-neg cells (isotype ratio of 17.5; p<0.01) (Fig. 2). These results establish that GATA1s represses cell differentiation towards the megakaryocytic lineage in DS AMkL.

### Down-regulation of GATA1s in CMK Cells Increases Cytotoxicities to Ara-C, Daunorubicin, and VP-16

**Figure 3:**
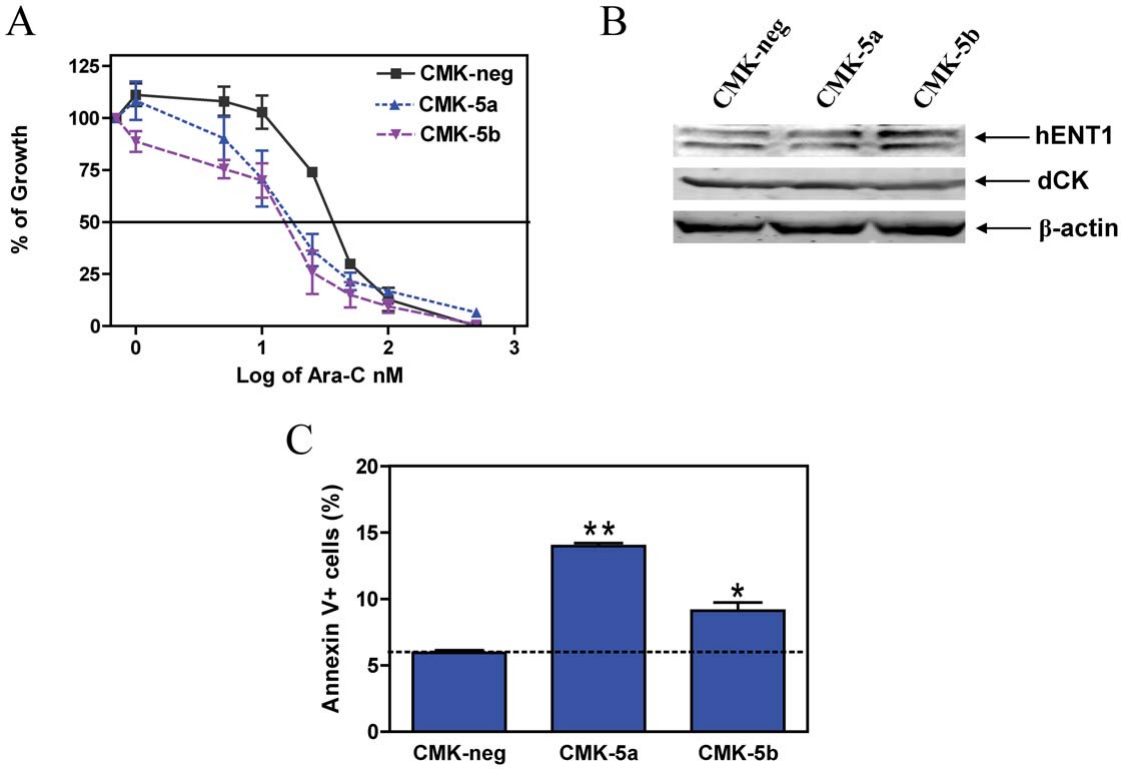
The effects of GATA1s on ara-C sensitivity in CMK cells. **Panel A**: The CMK-5a, -5b, and -Neg cells were cultured in complete medium with dialyzed fetal bovine serum in 96-well plates at a density of 8×10^4^ cells/ml, with a range of concentrations of ara-C at 37°C, and viable cell numbers were determined using the Cell Titer-blue reagent and a fluorescent microplate reader. The IC_50_ values were calculated as the concentrations of drug necessary to inhibit 50% growth compared to control cells cultured in the absence of drugs. The data are presented as mean values ± standard errors from at least 3 independent experiments. **Panel B**: Soluble proteins from the CMK-5a, -5b, and –neg sublines were subjected to Western blotting and probed by anti-hENT1, -dCK, or β-actin antibodies. **Panel C**: Basal apoptosis in the CMK-5a, -5b, and –neg cells was determined by annexin V/PI staining and flow cytometry analysis, as described in the Materials and Methods. * and ** indicate p<0.05 and 0.005, respectively.

To determine the role of GATA1s in ara-C, daunorubicin, and VP-16 sensitivities, *in vitro* drug cytotoxicities were determined in the CMK shRNA stable clones with decreased levels of GATA1s. Sensitivities to ara-C for the CMK-5a and CMK-5b cells, as reflected in IC_50_ values, were 2.1- and 2.5- fold, respectively, greater than that for the CMK-neg cells (Fig. 3A). Essentially the same results were obtained for daunorubicin and VP-16 in the shRNA stable clones (data not shown). This suggests that expression of GATA1s confers resistance to standard chemotherapeutic drugs in DS AMkL rather than enhancing their sensitivities. Hence, loss of GATA1 rather than expression of GATA1s is likely an important factor accounting for the enhanced chemosensitivity in DS AMkL compared to non-DS AML, leading to high EFS rates.[16]

To begin to determine the molecular mechanism(s) responsible for the changes in ara-C sensitivity accompanying decreased expression of GATA1s, we used real-time RT-PCR to quantify transcript levels for genes encoding ara-C metabolizing enzymes, *dCK* and *CDA*, and for *hENT-1*, which encodes the major membrane nucleoside transporter of ara-C. Interestingly, there were no consistent patterns for transcript levels for *dCK* and *hENT-1* accompanying decreased GATA1s (data not shown). *CDA* transcripts were not detectable in the CMK stable clones (data not shown). Similar results were obtained for the protein levels for dCK and hENT1 (Fig. 3B). These results clearly demonstrate that the effects of GATA1s on ara-C sensitivity in DS AMkL must involve mechanism(s) unrelated to altered expression of major genes involved in ara-C transport and metabolism.

The lack of changes in expression of hENT1, CDA and dCK among the CMK-5a, -5b, and –neg stable clones led to a hypothesis that GATA1s can modulate ara-C sensitivity in CMK cells by regulating expression of genes related to apoptosis. Further, the increased sensitivities to daunorubicin and VP-16 of the CMK-5a and -5b stable clones compared to the CMK-neg cells also support these notions. To test this possibility, baseline apoptosis in the CMK-5a, -5b, and –neg clones was determined by Annexin-V FITC/PI staining and flow cytometry analysis. Basal apoptosis in both CMK-5a and -5b cells was significantly higher (2.3-fold and 1.5-fold, respectively, p<0.05) than that in the CMK-neg cells and was inversely proportional to levels of GATA1s (Fig. 3C). These results strongly suggest that downregulation of GATA1s lowers the apoptotic threshold contributing to the increased ara-C sensitivity in CMK cells.

**Figure 4:**
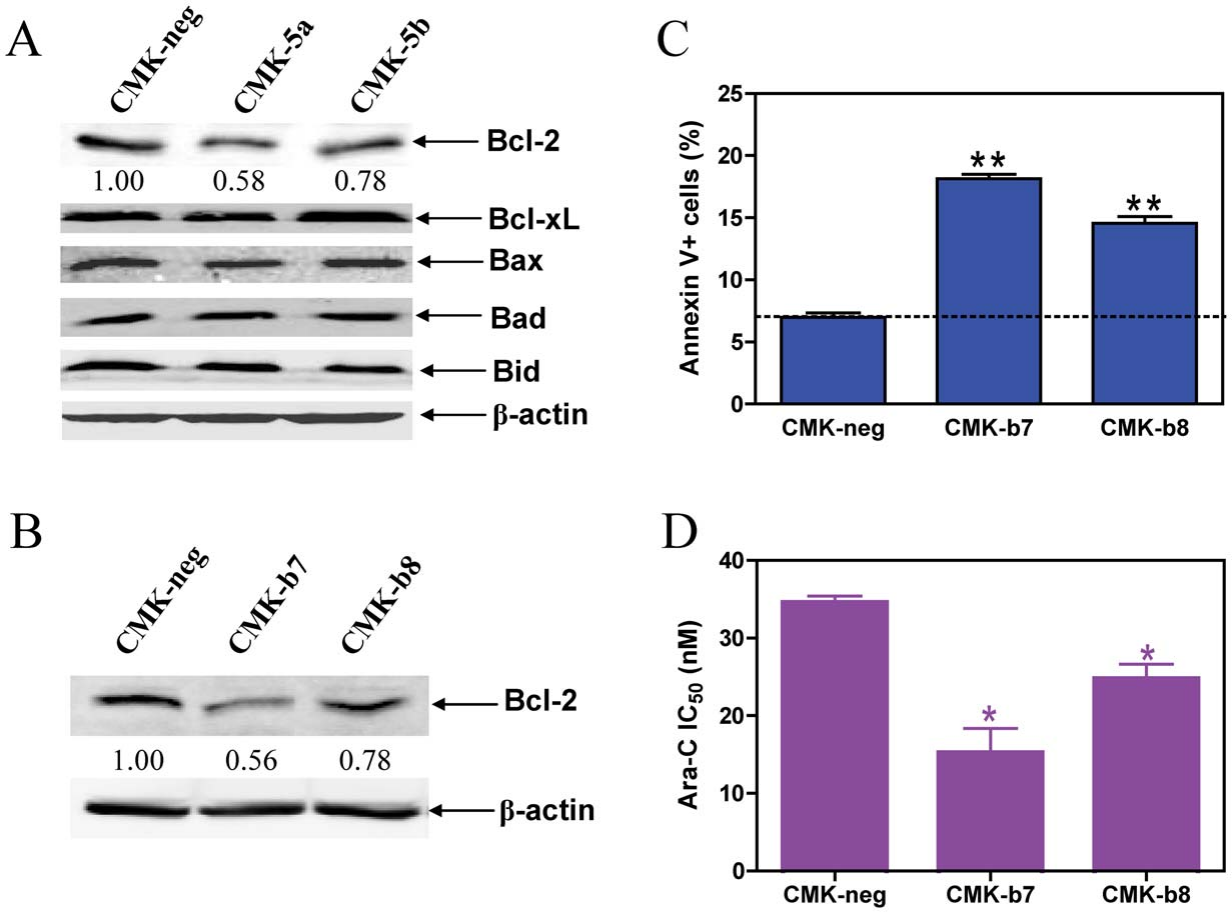
GATA1s exerts its effects on ara-C sensitivity through modulating Bcl-2 protein levels in CMK cells. **Panel A**: Soluble proteins from the CMK-5a, -5b, and –neg sublines were subjected to western blotting and probed by anti-Bcl-2, -Bcl-xL, -Bax, -Bad, -Bid, or β-actin antibodies. **Panel B**: Expression of Bcl-2 in two selected subclones, CMK-b7 and –b8, in comparison to the negative control (CMK-neg) was verified by Western blotting. **Panel C**: Basal apoptosis in the CMK-b7, -b8, and –neg cells was determined by annexin V/PI staining and flow cytometry analysis, as described in the Materials and Methods. **Panel D**: The CMK-b7, -b8, and -Neg cells were cultured in complete medium with dialyzed fetal bovine serum in 96-well plates at a density of 8×10^4^ cells/ml, with a range of concentrations of ara-C at 37°C, and viable cell numbers were determined using the Cell Titer-blue reagent and a fluorescent microplate reader. The IC_50_ values were calculated as the concentrations of drug necessary to inhibit 50% growth compared to control cells cultured in the absence of drugs. The data are presented as mean values ± standard errors from at least 3 independent experiments. * and ** indicate p<0.05 and 0.005, respectively.

Since chemotherapy drugs induce apoptosis in cancer cells primarily through the intrinsic apoptotic pathway, we performed Western blotting to identify changes in apoptotic factors potentially involved in the increased ara-C sensitivity associated with down-regulation of GATA1s. Interestingly, substantially decreased levels of the anti-apoptotic Bcl-2 protein were detected in the CMK-5a and -5b stable clones (decreases of 42% and 22%, respectively, relative to CMK-neg), while protein levels for other Bcl-2 family members, including Bcl-xL, Bad, Bax and Bid, were not altered (Fig. 4A). Surprisingly, transcript levels for *Bcl-2* were increased approximately 2-fold in the CMK-5a and -5b clones compared to the CMK-neg cells despite *decreased* levels of the protein (data not shown). These results suggest that the net impact of GATA1s on Bcl-2 is post-transcriptional, although the mechanism responsible for this effect is unclear.

To determine if Bcl-2 was the causal factor for the enhanced ara-C sensitivities in the CMK-5a and -5b clones compared to the CMK-neg cells, shRNA knockdown of *Bcl-2* gene was performed in CMK cells (Fig. 4B). As expected, decreased expression of Bcl-2 in CMK cells resulted in significantly increased basal apoptosis (Fig. 4C) and ara-C sensitivity (Fig. 4D) compared to the CMK-neg cells.

### Identification of GATA1s Responsive Genes in CMK cells

**Figure 5:**
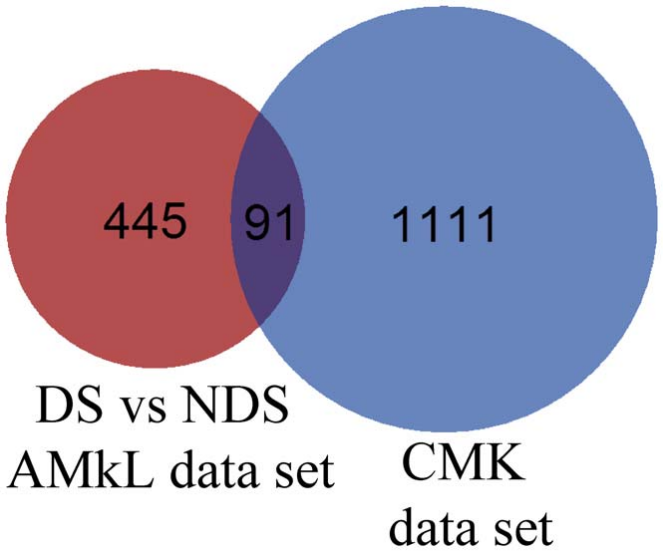
Identification of overlapping genes between the CMK microarray gene set and the previously reported microarray gene set derived from a comparison between DS and non-DS AMkL patient samples. For each gene set, differentially expressed probes were cross referenced to Entrez Gene IDs. That provided 1111 and 445 Entrex IDs for the CMK gene set and the DS vs non-DS AMkL gene set, respectively. The common set between the 2 groups was found and shown in a Venn representation.

To further investigate the molecular mechanisms that underlie the effects of GATA1s on DS AMkL cell proliferation, differentiation, and sensitivitiy to ara-C, we performed oligonucteotide microarray analyses on RNAs from the CMK-5a or CMK-5b, and CMK-neg cells. Average log ratios representing the differences in expression between the *GATA1* shRNA clone (CMK-5a or -5b) and the CMK-neg samples were derived for each array feature by combining replicate array data, using the error-weighted algorithm of Rosetta Resolver®.[21] Differentially expressed genes were identified by their p-values, calculated with the Resolver error-model and the replicate data. Using a p-value≤0.001 and a minimum ± 2.0 fold change as cutoffs, 1782 differentially expressed features (probes) were identified (Table S2), with a false discovery rate <1%. These probe IDs were then cross referenced to Entrez Gene IDs. That provided 1111 Entrez IDs. When these Entrez IDs were compared to those from our previously reported microarray gene set derived from a comparison between DS and non-DS AMkL patient samples, 91 Entrez IDs were found to be common in both gene sets (Figure 5). These results strongly suggest that GATA1s may contribute to the DS AMkL phenotype through regulating its responsive genes.

**Figure 6:**
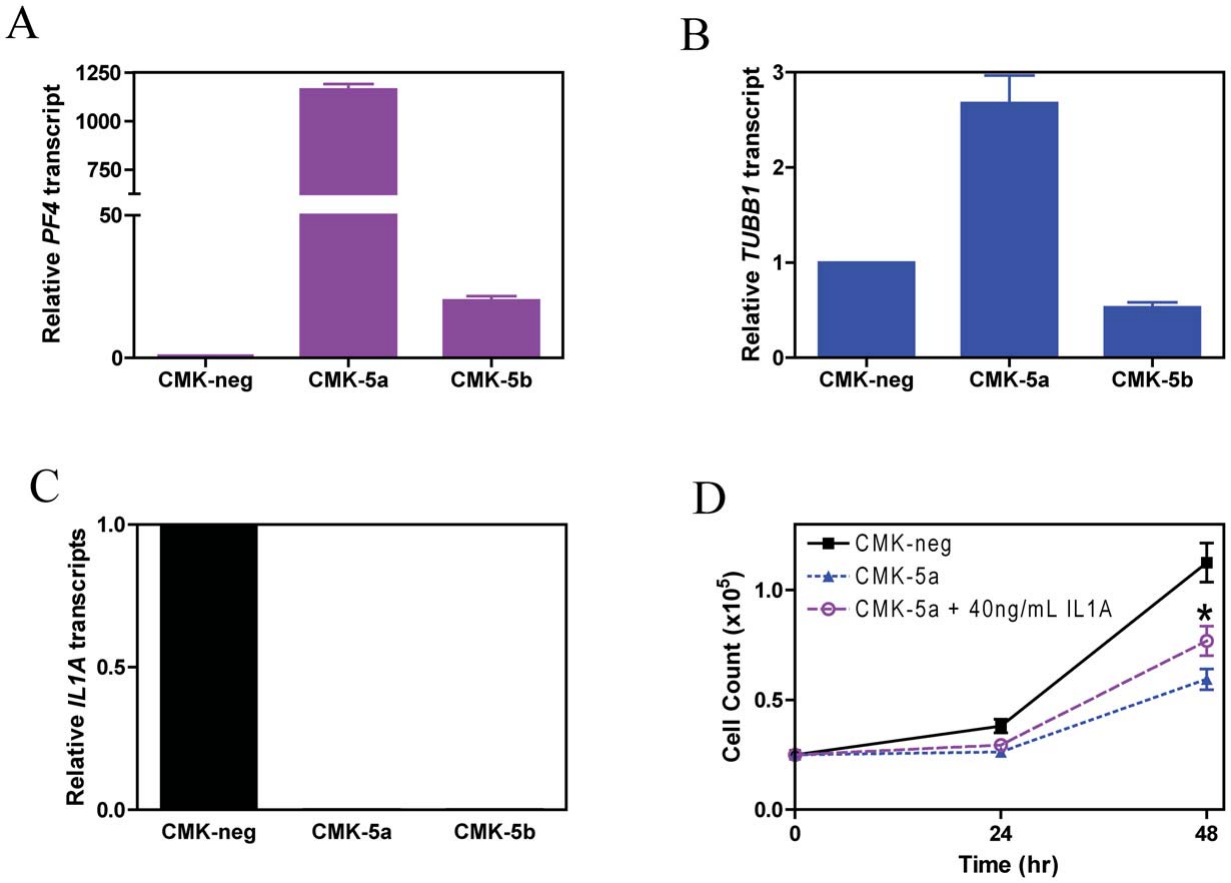
Down-regulation of GATA1s affects *PF4*, *TUBB1*, and *IL1A* expression in CMK cells. **Panels A–C:** Transcript levels for *PF4* (panel A), *TUBB1* (panel B), and *IL1A* (panel C) in the CMK-5a, -5b, and –neg cells were quantified by real-time RT-PCR as described in the Materials and Methods and results were expressed as mean values ± standard errors from 3 independent experiments using the same cDNA preparation and normalized to *GAPDH*. **Panel D:** CMK-5a cells were incubated with or without 40 ng/mL recombinant IL1α protein for up to 48 h and counted in triplicate every 24 h. * indicates p<0.05.

Interestingly, a large number of these 1782 differentially expressed probes are involved with cell differentiation (Table S3), proliferation (Table S4), and death (Table S5). Among the genes relevant to cell differentiation and proliferation, *IL1A* (*interleukin 1 alpha), PF4 (platelet factor 4),* and *TUBB1* (*tubulin beta 1),* were of particular interest. *IL1A* encodes a cytokine which enhances proliferation of megakaryocytic progenitors.[25] *PF4* and *TUBB1* encode proplatelet formation proteins and increased expression of these genes would suggest differentiation toward the megakaryocytic lineage. Oligonucleotide microarray analysis revealed 11.0- and 2.4-fold increased expression of *PF4* and *TUBB1*, respectively, and a 2.1-fold decreased expression of *IL1A* in the CMK-5a and -5b clones compared to the CMK-neg cells. Real-time RT-PCR confirmed a nearly complete lack of *IL1A* expression and substantially increased expression of *PF4* in the CMK-5a and -5b clones compared to the CMK-neg cells (Fig. 6A&C). Substantially increased expression of *TUBB1* was also detected by real-time RT-PCR in the CMK-5a but not in the CMK-5b clone, compared to the CMK-neg cells (Fig. 6B). As expected, cell proliferation was significantly increased when CMK-5a cells were treated with 40 ng/mL recombinant IL1α (Fig. 6D). Thus, GATA1s may impact proliferation and megakaryocytic differentiation of CMK cells by regulating the expression of relevant genes.

### IL1A is a Direct GATA1s Target Gene

**Figure 7:**
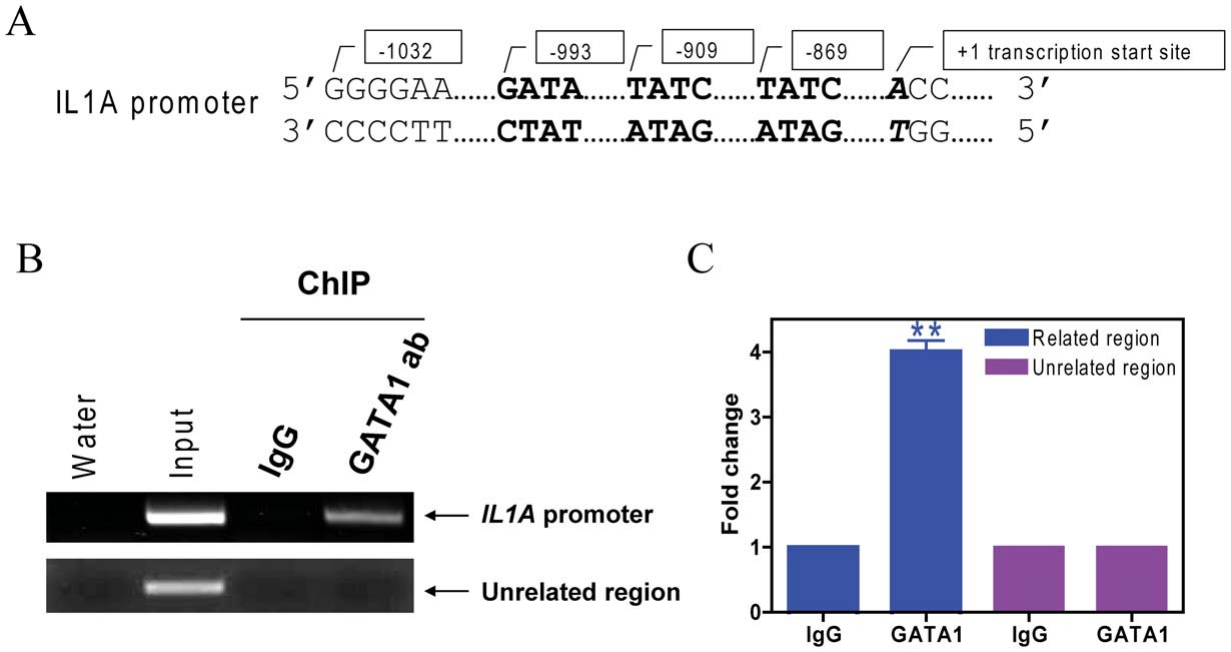
*IL1A* is a bona fide GATA1s target gene in DS AMkL. *In vivo* binding of GATA1s to the putative GATA1 binding sites located in the upstream region of the *IL1A* gene (**panel A**) in CMK cells was determined by ChIP assays with use of regular PCR (**panel B**) and real-time PCR (**panel C**), as described in Materials and Methods. ** indicates p<0.005.

It was of interest to establish whether *IL1A* is a direct GATA1s target gene. Three putative GATA1 binding sites (GATA) located at positions −993 to −990, −909 to −906, and −869 to −866 (relative to the transcription start site, based on *IL1A* mRNA sequence NM_000575) are present in the *IL1A* promoter region (Fig. 7A). In CMK cells, GATA1s directly bound to the GATA1 binding sites of *IL1A in vivo* when assayed by ChIP with either regular PCR (Fig. 7B) or real-time PCR (Fig. 7C). These results demonstrated that *IL1A* is a *bona fide* GATA1s target in CMK cells. Thus, GATA1s likely exerts its functions by directly regulating expression of genes important for cell proliferation, survival, and megakaryocytic differentiation.

## Discussion

The *GATA1* gene encodes a zinc finger transcription factor that is essential for normal erythroid and megakaryocytic differentiation.[13], [26]–[29] Acquired somatic mutations in exon 2 of *GATA1* gene have been consistently detected in nearly all DS TMD and AMkL cases, whereas none were detected in non-DS AML and non-AMkL DS leukemia except for rare cases,[10]–[12], [30]–[32] highlighting their critical roles in leukemogenesis in DS. The net effect of these mutations is the introduction of stop codons and synthesis of GATA1s (40 kDa), initiated from a downstream initiation site and distinguishable from GATA1 (50 kDa).[12] Both GATA1s and GATA1 show similar DNA binding abilities and interact with partner proteins, such as FOG1, although GATA1s exhibits altered transactivation capacity due to the loss of the *N*-terminal activation domain.[12] Lineage selective knockdown of *GATA1* in megakaryocytes has been reported to cause increased megakaryocyte proliferation coupled with impaired maturation in mice.[27]

It is believed that the presence of constitutional chromosome 21 disturbs fetal hematopoiesis promoting the acquisition of *GATA1* mutations.[33], [34] As a consequence, *GATA1* mutations would promote accumulation of poorly differentiated megakaryocytic precursors and would represent initiating or early “genetic hits” in a multi-step process of leukemogenesis initiated prenatally.[35] One mechanism by which those effects can be explained is that GATA1s fails to repress certain genes normally regulated by GATA1.[36] However, the possibility that expression of GATA1s in DS AMkL may itself be causal, due to its unique function in regulating specific genes, cannot be excluded. To date, the possibly unique role of GATA1s in promoting leukemogenesis and chemotherapy sensitivity in a human DS AMkL model has not been considered.

In this report, we established that GATA1s has novel biological functions in relation to DS AMkL leukemogenesis and responses to therapy. Our approach involved lentiviral shRNA knockdown of the *GATA1* gene in a human DS AMkL cell line, CMK, which harbors a mutated *GATA1* gene and expresses only GATA1s. Interestingly, down-regulation of GATA1s in CMK cells resulted in impaired cell proliferation and increased differentiation toward the megakaryocytic lineage to extents that paralleled the decreased levels of GATA1s seen in the stable clones (Fig. 1 and Fig. 2). The increase in megakaryocytic differentiation of the CMK-5a and CMK-5b cells was further supported by the substantially induced expression of proplatelet formation genes, such as *PF4* and *TUBB1* (Fig. 6). In addition, down-regulation of GATA1s was accompanied by both up- and down-regulation of a large number of genes, including genes related to cell proliferation and cell differentiation, such as *IL1A.* Previous studies have established that interleukin 1 enhances proliferation of megakaryocytic progenitors.[25], [37] Our finding that GATA1s promotes cell proliferation and represses differentiation toward megakaryocytic lineage in DS suggests that expression of GATA1s in itself could be a causal factor in leukemogenesis in DS.

EFS rates for DS patients with AMkL are significantly higher than non-DS AML patients, and in particular, non-DS AMkL patients,[2]–[5] suggesting that the presence of GATA1s may contribute to the high EFS rates of DS AMkL patients. Our previous study suggested that loss of GATA1 may contribute to the enhanced chemotherapy responses of DS AMkL compared to non-DS AML.[38] However, it is not clear whether expression of GATA1s itself contributes to the increased chemotherapy sensitivities, as seen in DS AMkL patients, compared to non-DS AML patients. In the present study, we found that down-regulation of GATA1s in CMK cells resulted in significantly increased *in vitro* sensitivities to ara-C, daunorubicin, and VP-16 compared to that in the negative control, suggesting that expression of GATA1s can confer resistance to chemotherapy in DS AMkL.

Accordingly, expression of GATA1s by itself is *unlikely* to explain the enhanced chemotherapy responses in DS AMkL compared to non-DS AML. Rather, expression levels of GATA1s may represent an important biomarker related to chemotherapy sensitivity or resistance in DS AMkL. Loss of GATA1 may represent a significant contributing factor accounting for the increased chemotherapy sensitivity of DS AMkL. The role of GATA1s in chemosensitivity only applies to the DS AML group itself (based on the lack of expression in non-DS AML) and its relative impact may be modest. Nonetheless, assessing the role of GATA1s expression in DS AMkL may identify a small subset of DS patients with refractory or relapsed disease.[39] A study of DS patients with TMD reported that differences in GATA1s levels were associated with the risk of progression to myeloid leukemias, supporting the notion that GATA1s may impact the clinical features of DS leukemia cases. [40]

What are the molecular mechanism(s) responsible for the effects of GATA1s on ara-C sensitivity? Our real-time RT-PCR assays showed no significant differences in *dCK*, *CDA*, and *hENT-1* transcript levels among the stable clones (data not shown). However, significantly increased basal apoptosis in the CMK-5a and -5b clones was detected compared to that in the CMK-neg cells. This was accompanied by decreased levels of Bcl-2 protein, which was certainly responsible for the increased basal apoptosis and increased ara-C sensitivities in the CMK-5a and -5b clones, as demonstrated by our lentiviral shRNA knockdown experiments. Surprisingly, down-regulation of GATA1s resulted in increased *Bcl-2* transcripts in the GATA1 shRNA stable clones compared to the CMK-neg cells, suggesting that GATA1s must confer some means of posttranscriptional regulation of Bcl-2 expression in CMK cells. Current studies are underway to determine the mechanisms underlying the regulation of Bcl-2 expression by GATA1s in CMK cells.

To summarize, our findings suggest that GATA1s has unique functions in DS AMkL. GATA1s appears likely to facilitate DS leukemogenesis and to confer resistance to chemotherapy by promoting proliferation and survival, and by repressing differentiation towards the megakaryocytic lineage, potentially by regulating expression of Bcl-2 and other relevant genes.

## Supporting Information

Figure S1 **Sequences for the negative control, GATA1, and Bcl-2 shRNAs.**
(DOC)

Table S1 **Summary of primers used for real-time RT-PCR in this study.**
(XLS)

Table S2 **GATA1s responsive probes identified by microarray analysis.**
(XLSX)

Table S3 **GATA1s responsive probes related to cell differentiation.**
(XLSX)

Table S4 **GATA1s responsive probes related to cell proliferation.**
(XLSX)

Table S5 **GATA1s responsive probes related to cell death.**
(XLSX)
