# Supplementary material for: A Unique Role of GATA1s in Down Syndrome Acute Megakaryocytic Leukemia Biology and Therapy
Source: PLoS One. 2011 Nov 16;6(11):e27486. doi: 10.1371/journal.pone.0027486 (PMC3217966; doi:10.1371/journal.pone.0027486)
Supplement: Figure S1 — Sequences for the negative control, GATA1, and Bcl-2 shRNAs. (DOC) [file pone.0027486.s001.doc]

**Figure S1. Sequences for the negative control, GATA1, and Bcl-2 shRNAs.**

**Negative control shRNA**

SHC002V

Sequence: CCGGCAACAAGATGAAGAGCACCAACTCGAGTTGGTGCTCTTCATCTTGTTGTTTTT

***GATA1* shRNA**

TRCN0000019223

Sequence: CCGGCCCTCAATTCAGCAGCCTATTCTCGAGAATAGGCTGCTGAATTGAGGGTTTTT

***BCL2* shRNA**

TRCN0000010303

Sequence: CCGGTGGATGACTGAGTACCTGAACCTCGAGGTTCAGGTACTCAGTCATCCATTTTTG
